# Variation in Human Papillomavirus Vaccination Effectiveness in the US by Age at Vaccination

**DOI:** 10.1001/jamanetworkopen.2022.38041

**Published:** 2022-10-21

**Authors:** Didem Egemen, Hormuzd A. Katki, Anil K. Chaturvedi, Rebecca Landy, Li C. Cheung

**Affiliations:** 1 Division of Cancer Epidemiology and Genetics, National Cancer Institute, National Institutes of Health, US Department of Health and Human Services, Bethesda, Maryland

## Abstract

This survey study assesses the status and timing of HPV vaccination as self-reported by female participants in the National Health and Nutrition Examination Survey from 2011 to 2018.

## Introduction

The Centers for Disease Control and Prevention Advisory Committee on Immunization Practices (ACIP) recommends routine human papillomavirus (HPV) vaccination at ages 9 to 12 years^1^ as protection against HPV types 16 and 18 (HPV-16/18), the genotypes in 70% of cervical cancers. Although the importance of vaccination uptake is well known, vaccination often occurs later than recommended.^2^ Recent studies have documented the effectiveness of early-age vaccination for reducing cervical precancer and cancer incidence.^3,4^ We used data from the National Health and Nutrition Examination Survey (NHANES)^5^ to estimate the proportion of female individuals who were vaccinated before sexual debut, assess the association of delayed vaccination with HPV-16/18 prevalence, and quantify potential racial and ethnic disparities in timely vaccination.

## Methods

NHANES is a biennial cross-sectional multistage probability sample representing the noninstitutionalized civilian US population.^5^ All NHANES participants or their guardians provided written informed consent. This study was deemed exempt from review by the National Cancer Institute Institutional Review Board because it used deidentified data sets. We followed the AAPOR reporting guideline. NHANES variables and the analytic approach are described in the eMethods in the Supplement.

Participants self-reported race and ethnicity, HPV vaccination, and sexual history and underwent vaginal HPV testing. Using responses to NHANES cycles 2011 to 2018, we identified female individuals who were 26 years or younger in 2006 (when HPV vaccination was introduced) and eligible for either routine (aged 9-12 years) or catch-up (aged 13-26 years) vaccination per ACIP recommendations.^1^ We compared HPV-16/18 prevalence among unvaccinated participants, those vaccinated before sexual debut (predebut group), and those vaccinated after (postdebut group) sexual debut. In participants eligible for routine vaccination (RV), we estimated vaccine uptake and proportion vaccinated by racial and ethnic subgroups.

Data were analyzed from September 2021 to March 2022 using R, version 3.6.0, “survey” package. A 2-sided *P* = .05 indicated significance.

## Results

Among the 4727 female individuals (mean [SD] age, 17.9 [0.2] years) ever eligible for vaccination, cervical HPV-16/18 prevalence decreased from 6% (95% CI, 4%-7%) in the unvaccinated group to 3% (95% CI, 1%-6%) in the postdebut group and less than 1% (95% CI, <1%-1%) in the predebut group (Figure). HPV-16/18 prevalence was 89% (*P* < .001) lower in the predebut group but only 41% (*P* = .29) lower in the postdebut group compared with the unvaccinated group. Compared with postdebut vaccination, predebut vaccination was associated with an 82% (*P* = .08) reduction in HPV-16/18 prevalence.

**Figure.**
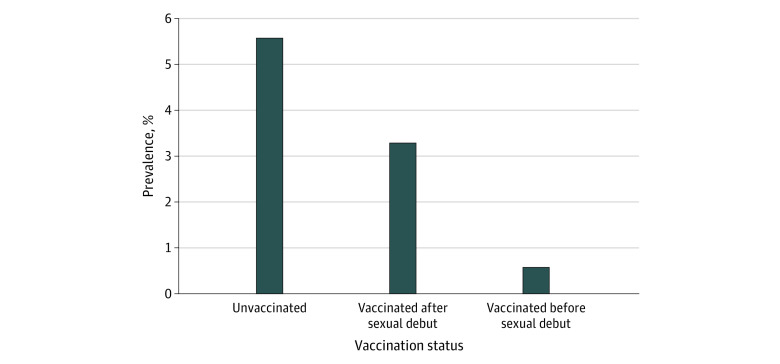
Weighted Prevalence of Human Papillomavirus Types 16 and 18 (HPV-16/18) Infections in Vaccine-Eligible Female Individuals HPV vaccination status was identified from National Health and Nutrition Examination Survey questionnaire responses on vaccination status, age of vaccination, and age of sexual debut. Individuals who were missing either vaccination status or HPV-16/18 test results were excluded.

Only 38% of ever-eligible participants were vaccinated, increasing to 56% when restricted to those eligible for RV. However, only 21% (95% CI, 14%-28%) of vaccinated, RV-eligible female adults reported receiving their first dose by age 12 years per ACIP recommendations (mean age at first vaccination dose, 14.5 [95% CI, 14.1-14.8] years). Among these adults, 33% were vaccinated before and 23% after sexual debut. Thus, 41% of vaccinated participants received postdebut vaccination.

Differences by race and ethnicity were negligible (6% of participants were Asian; 20%, Hispanic; 14%, non-Hispanic Black; 55%, non-Hispanic White; and 5%, other groups). Proportions of vaccinated Asian and White participants were slightly higher than those of Black and Hispanic participants (57% and 58% vs 54% and 52%; *P* = .22). Similar proportions of participants were vaccinated before sexual debut (32%-35%).

## Discussion

This study highlights the importance of timely vaccination against HPV, particularly before sexual debut. The findings confirm previous reports that many female individuals receive HPV vaccination after the ACIP-recommended ages.^2^ Vaccination by age 12 years is estimated to prevent most lifetime cervical cancers caused by HPV-16/18, but this benefit decreases by one-fourth as vaccination age increases to 16 years.^6^

A study limitation is that NHANES 2011 to 2018 data may be subject to recall bias. Further studies are needed to check whether these patterns persist in the 2020s, particularly as the COVID-19 pandemic may have impeded vaccine uptake. To ensure maximum effectiveness from vaccination, pediatricians may stress the importance of timely HPV vaccination.

## Notes

Supplement. **eMethods.** NHANES Survey and Variables, Literature Review, and Methods
eReferences
