# Supplementary material for: Variation in Human Papillomavirus Vaccination Effectiveness in the US by Age at Vaccination
Source: JAMA Netw Open. 2022 Oct 21;5(10):e2238041. doi: 10.1001/jamanetworkopen.2022.38041 (PMC9587484; doi:10.1001/jamanetworkopen.2022.38041)
Supplement: Supplement. — eMethods. NHANES Survey and Variables, Literature Review, and Methods eReferences [file jamanetwopen-e2238041-s001.pdf]

Supplementary Online Content

Egemen D, Katki HA, Chaturvedi AK, Landy R, Cheung LC. Variation in human papillomavirus vaccination effectiveness in the US by age at vaccination. *JAMA Netw Open*. 2022;5(10):e2238041. doi:10.1001/jamanetworkopen.2022.38041

eMethods. NHANES Survey and Variables, Literature Review, and Methods

eReferences

This supplementary material has been provided by the authors to give readers additional information about their work.

© 2022 Egemen D et al. *JAMA Network Open.*

eMethods. NHANES Survey and Variables, Literature Review, and Methods

NHANES SURVEY AND VARIABLES

The National Health and Nutrition Examination Survey (NHANES) is a periodic cross-sectional survey conducted every 2 years by the National Center for Health Statistics (NCHS), Centers for Disease Control and Prevention (CDC). This survey, which aims to assess the health and nutritional status of adults and children in the United States, uses a complex, multistage probability design to represent the resident civilian, non-institutionalized US population. The survey was administered in 2 parts: a home interview and a medical exam through a mobile examination center (MEC). NHANES protocol was approved by the US Centers for Disease Control and Prevention National Center for Health Statistics Ethics Review Board, and all participants provided written informed consent, or assent was obtained from participants or their guardians, respectively. This study was exempt from the NCI institutional review board review because it used publicly de-identified data sets. Further details on the NHANES survey design can be found in related documentation.[1] This analysis required restricted use data accessed through the Research Data Center (RDC), and our study followed the American Association for Public Opinion Research (AAPOR) reporting guideline.[2]

In our analysis, we used 4 cycles of NHANES (2011-2012, 2013-2014, 2015-2016, and 2017-2018). The combined data set treats each NHANES cycle as a separate strata and weighting to the 2011-2018 US population is achieved by dividing the original weight by 4. The variables included in this analysis with the corresponding survey questions are as follows:

- <u>Ever had vaginal sex and Age of sexual debut:</u> All questions regarding sexual behavior were self-administered in a private room during the medical examination part of the

© 2022 Egemen D et al. *JAMA Network Open.*

survey, using the Audio Computer-Assisted Self Interview (ACASI) system. Female participants were asked "Have you ever had vaginal sex, also called sexual intercourse with a man? This means a man's penis in your vagina." and "How old were you the first time you had any kind of sex, including {vaginal, anal, oral}?" For the latter question, only the kinds of sex in which the participant reported having on earlier questions were displayed. The response was coded as an integer age.

- <u>Ever receive HPV vaccine and age of first dose of HPV vaccine:</u> Questions regarding immunization were asked in the home, by trained interviewers, using the Computer-Assisted Personal Interview (CAPI) system. The questions participants answered were "Human Papillomavirus (HPV) vaccine is given to prevent cervical cancer in girls and women. The HPV vaccines available are called Ceravix, Gardasil, and Garadsil 9. It is given in 3 separate doses over a 6 month period. Have you ever received one or more doses of the HPV vaccine?" and "How old were you when you received your first dose of {Cervarix/Gardasil/Gardasil 9/Gardasil or Gardasil 9/the vaccine}?" The response was coded as an integer age.
- <u>HPV 16/18 infection:</u> As part of NHANES, female participants underwent a vaginal examination in which cervical samples were collected. The samples were evaluated by Roche Linear Array HPV Genotyping test and reported 37 high-risk HPV types as separate channels. In our analysis, we combined HPV 16 and HPV18 results (each reported as positive or negative) to create a variable indicating whether an individual was infected with either HPV 16 or 18.
- <u>HPV vaccination status:</u> We created a 3-level categorical variable as; unvaccinated, vaccinated before sexual debut, and vaccinated after sexual debut. Individuals who

responded “no” to ever-receiving an HPV vaccine were classified as unvaccinated. Among individuals who reported “yes” to receiving the HPV vaccination, we classified as vaccinated before sexual debut if they reported no to vaginal sex or if they reported yes to vaginal sex but the age of sexual debut was greater than the age of HPV vaccination. Individuals who reported yes to HPV vaccination, yes to vaginal sex, and had an age of sexual debut less than or equal to the age of HPV vaccination were classified as vaccinated after sexual debut. The vaccinated after sexual debut classification may include individuals for whom vaginal sexual debut occurred after HPV vaccination. All other individuals were classified with an unknown vaccination status.

- Race/ethnicity: This variable was derived from participant responses to the survey questions on race and Hispanic origin. The questionnaire was administered in both English and Spanish. The specific questions are
  - “What race(s) do you consider {yourself/NAME} to be? Please select one or more.” (The multiple-choice answers are: 1) American Indian or Alaskan Native, 2) Asian, 3) Black or African American, 4) Native Hawaiian or Pacific Islander, 5) White, 6) Other), “Do any of the groups on this card represent {your/NAME’s} national origin or ancestry?”.
  - “[Do you/Does NAME] consider [yourself/himself/herself] to be Hispanic, Latino, or of Spanish origin?”, If necessary, an additional question "Where {do your/do his/do her} ancestors come from?" was asked. Answers of Puerto Rican, Cuban/Cuban American, Dominican Republic, Mexican/Mexican American, Central/South American, Other Latin American, and Other Hispanic or Latino were classified as of Hispanic origin.

© 2022 Egemen D et al. *JAMA Network Open.*

These variables are combined into an NHANES self-reported race/ethnicity variable with categories of “Mexican American”, “Non-Hispanic Asian”, “Non-Hispanic Black”, “Non-Hispanic White”, “Other Hispanic”, “Other Race – Including Multi-Racial”. In our analysis, we recoded classifications as “Hispanic” (combining Mexican American and Other Hispanic), “Non-Hispanic Asian”, “Non-Hispanic Black”, “Non-Hispanic White”, “Other Race – Including Multi-Racial”.

LITERATURE REVIEW

Various studies and trials have examined the efficacy of the HPV vaccine by age of vaccination.[3-4-5] Using the Costa Rica Vaccine Trial (CVT), Herrero et al.[6] reported no change in vaccine efficacy according to age of vaccination when administered in women who were HPV 16/18-negative at enrollment; however, efficacy decreased as the age of vaccination increased within the full cohort that could include women who were HPV 16/18-positive. Using electronic health records from Kaiser Permanente Northern California, Castle et al. examined the three-year risk of precancer among women aged 21-24 years with low-grade cytology results.[7] He found that women vaccinated before age 18 had lower precancer risk than unvaccinated women and women vaccinated at older ages. Herweijer et al. reported similar findings in Sweden, with women vaccinated before age 17 years having reduced precancer rates compared to unvaccinated women and women vaccinated at older ages.[8] In a microsimulation modeling study, Demarteau et al. estimated the number of preventable cervical cancers through HPV vaccination and showed that as the age of vaccination increases, fewer cervical cancers can be preventable.[9] Falcaro et al. examined the effect of offering HPV vaccination in the United Kingdom and found that women who were younger at the time of vaccine offer had greater reduction in cervical cancer rates

© 2022 Egemen D et al. *JAMA Network Open.*

compared to earlier cohorts not eligible for the HPV vaccine.[10] In contrast to prior publications, we examine HPV 16/18 prevalence in individuals who were unvaccinated vs. vaccinated after sexual debut vs. vaccinated before sexual debut. Among individuals eligible for vaccination by age 12 years (ACIP recommends routine HPV vaccination at ages 9 to 12 years.[11]), we estimated the proportions who were unvaccinated, received vaccination before sexual debut, and received vaccination after sexual debut in the US population.

In our analysis, the maximum time from vaccination until assessment for HPV16/18 is 12 years. Several prior studies have examined the long-term effect of HPV vaccination. Porras et al.[12] found ~95% reduction against HPV16/18 precancers at year 11 in the Costa Rica Vaccine Trial. Lehtinen et al.[13] reported similar long-term efficacy after 10 years in the PATRICIA trial. Artemchuk et al.[14] reported that anti-HPV 16/18 antibody levels remained stable after 12 years in the Finnish Maternity Cohort.

METHODS

We used NHANES cycles 2011-12, 2013-14, 2015-16, and 2017-18. All portions of NHANES 2011-16 used in the analysis was available as of November 2018. For the 2017-18 NHANES cycle, immunization and demographic data was released for public access in February 2020, sexual behavior was released under restricted use in July 2020, and type-specific HPV genotyping lab results were completed and released under restricted use in December 2020. To access the NHANES 2017-18 restricted-use data and the restricted use data for NHANES 2011-18 respondents aged 14-18 years, we submitted a research proposal (approved in 2021), visited a secure Research Data Center (RDC) to conduct the analysis (completed Spring 2022), and received approval from the RDC to disseminate the results (completed April 2022).

© 2022 Egemen D et al. *JAMA Network Open.*

Using NHANES 2011-2018, we identified individuals who were ever eligible for vaccination (age 26 years or younger in 2006, the first year in which the HPV vaccine was available). This group was further stratified into individuals who were ever-eligible for routine vaccination (age 12 years or younger in 2006) and individuals who were only eligible for catch-up vaccination (ages 13-26 years in 2006).

For all females ever-eligible for vaccination in NHANES 2011-18, we used the R *survey* package to estimate the weighted HPV 16/18 prevalence and standard errors for the three vaccination categories (unvaccinated, vaccinated before sexual debut/predebut group, and vaccinated after sexual debut/postdebut group). 95% confidence intervals are estimated by assuming asymptotic normality. Percent reductions were estimated using the prevalence estimates. To estimate p-values with and without adjustment for age, we fitted survey logistic regression models with and without age as a covariate. We considered the effects of other covariates (e.g., marital status and education level), but these variables did not add predictive value for HPV 16/18 prevalence beyond that provided by vaccination status and age.

For adult females ever-eligible for HPV vaccination and adult females ever-eligible for routine vaccination, we estimated the percent in each vaccination category, the weighted average age at vaccination, and the proportion of vaccinated individuals who received their first HPV dose by age 12. We further examined differences in these variables by racial and ethnicity.

© 2022 Egemen D et al. *JAMA Network Open.*

eReferences

1. Centers for Disease Control and Prevention (CDC). National Center for Health Statistics (NCHS). National Health and Nutrition Examination Survey Data. Hyattsville, MD: U.S. Department of Health and Human Services, Centers for Disease Control and Prevention, [2011-2018] [https://wwwn.cdc.gov/nchs/nhanes].
2. The American Association for Public Opinion Research. 2016. Standard Definitions: Final Dispositions of Case Codes and Outcome Rates for Surveys. 9th edition. AAPOR.
3. Munoz, N., Kjaer, S.K., Sigurdsson, K., et al., 2010 Mar 3. Impact of human papillomavirus (HPV)-6/11/16/18 vaccine on all HPV-associated genital diseases in young women. J. Natl. Cancer Inst. 102 (5), 325–339.
4. Kjaer, S.K., Sigurdsson, K., Iversen, O.E., et al., 2009 Oct. A pooled analysis of continued prophylactic efficacy of quadrivalent human papillomavirus (types 6/11/16/18) vaccine against high-grade cervical and external genital lesions. Cancer Prev. Res. (Phila.) 2 (10), 868–878.
5. Lehtinen, M., Paavonen, J., Wheeler, C.M., et al., 2012 Jan. Overall efficacy of HPV-16/18 AS04-adjuvanted vaccine against grade 3 or greater cervical intraepithelial neoplasia: 4-year end-of-study analysis of the randomized, double-blind PATRICIA trial. Lancet Oncol. 13 (1), 89–99.
6. Herrero R, Wacholder S, Rodríguez AC, et al. Prevention of persistent human papillomavirus infection by an HPV16/18 vaccine: a community-based randomized clinical trial in Guanacaste, Costa Rica. Cancer Discov. 2011;1(5):408-19.
7. Castle PE, Xie X, Xue X, et al. Impact of human papillomavirus vaccination on the clinical meaning of cervical screening results. Prev Med. 2019;118:44-50.

© 2022 Egemen D et al. *JAMA Network Open.*

8. Herweijer E, Sundström K, Ploner A, et al. Quadrivalent HPV vaccine effectiveness against high-grade cervical lesions by age at vaccination: A population-based study. Int J Cancer. 2016;138(12):2867-74.
9. Demarteau N, Kriekinge GV, Simon P. Incremental cost-effectiveness evaluation of vaccinating girls against cervical cancer pre- and post-sexual debut in Belgium. Vaccine. 2013;31(37): 3962-3971.
10. Falcaro M, Castanon A, Ndlela Busani, et al. The effects of the national HPV vaccination programme in England, UK, on cervical cancer and grade 3 cervical intraepithelial neoplasia incidence: a register-based observational study. The Lancet 2021;398(10316):2084-2092.
11. Meites E, Szilagyi PG, Chesson HW, et al. Human Papillomavirus Vaccination for Adults: Updated Recommendations of the Advisory Committee on Immunization Practices. *MMWR Morb Mortal Wkly Rep* 2019;68:698–702. doi:10.15585/mmwr.mm6832a3
12. Porras C, Tsang SH, Herrero R, et al. Efficacy of the bivalent HPV vaccine against HPV 16/18-associated precancer: long-term follow-up results from the Costa Rica Vaccine Trial. Lancet Oncol. 2020 Dec;21(12):1643-1652.
13. Lehtinen M, Lagheden C, Luostarinen T, et al. Ten-year follow-up of human papillomavirus vaccine efficacy against the most stringent cervical neoplasia end-point—registry-based follow-up of three cohorts from randomized trials. BMJ Open 2017;7:e015867 .

© 2022 Egemen D et al. *JAMA Network Open.*

14. Artemchuk H, Eriksson T, Poljak M, et al. Long-term Antibody Response to Human Papillomavirus Vaccines: Up to 12 Years of Follow-up in the Finnish Maternity Cohort. J Infect Dis. 2019 Jan 29;219(4):582-589.

© 2022 Egemen D et al. *JAMA Network Open.*
